# Shifting Regimes and Changing Interactions in the Lake Washington, U.S.A., Plankton Community from 1962–1994

**DOI:** 10.1371/journal.pone.0110363

**Published:** 2014-10-22

**Authors:** Tessa B. Francis, Elizabeth M. Wolkovich, Mark D. Scheuerell, Stephen L. Katz, Elizabeth E. Holmes, Stephanie E. Hampton

**Affiliations:** 1 University of Washington Tacoma, Puget Sound Institute, Tacoma, Washington, United States of America; 2 National Center for Ecological Analysis and Synthesis, University of California Santa Barbara, Santa Barbara, California, United States of America; 3 The Biodiversity Research Centre, University of British Columbia, Vancouver, British Columbia, Canada; 4 Fish Ecology Division, Northwest Fisheries Science Center, National Marine Fisheries Service, National Oceanic and Atmospheric Administration, Seattle, Washington, United States of America; 5 Channel Islands National Marine Sanctuary, National Ocean Service, National Oceanic and Atmospheric Administration, Santa Barbara, California, United States of America; 6 Conservation Biology Division, Northwest Fisheries Science Center, National Marine Fisheries Service, National Oceanic and Atmospheric Administration, Seattle, Washington, United States of America; UC Santa Cruz Department of Ecology and Evolutionary Biology, United States of America

## Abstract

Understanding how changing climate, nutrient regimes, and invasive species shift food web structure is critically important in ecology. Most analytical approaches, however, assume static species interactions and environmental effects across time. Therefore, we applied multivariate autoregressive (MAR) models in a moving window context to test for shifting plankton community interactions and effects of environmental variables on plankton abundance in Lake Washington, U.S.A. from 1962–1994, following reduced nutrient loading in the 1960s and the rise of *Daphnia* in the 1970s. The moving-window MAR (mwMAR) approach showed shifts in the strengths of interactions between *Daphnia*, a dominant grazer, and other plankton taxa between a high nutrient, *Oscillatoria*-dominated regime and a low nutrient, *Daphnia*-dominated regime. The approach also highlighted the inhibiting influence of the cyanobacterium *Oscillatoria* on other plankton taxa in the community. Overall community stability was lowest during the period of elevated nutrient loading and *Oscillatoria* dominance. Despite recent warming of the lake, we found no evidence that anomalous temperatures impacted plankton abundance. Our results suggest mwMAR modeling is a useful approach that can be applied across diverse ecosystems, when questions involve shifting relationships within food webs, and among species and abiotic drivers.

## Introduction

One of the most important challenges facing ecologists is specifying how global change will affect community stability and the production of associated critical ecosystem services. Community stability is mediated by species interactions, which are sensitive to changing environmental conditions [1], [2], and therefore estimating the effects of environmental drivers on food web dynamics is critical for understanding how anthropogenic forces have altered ecosystems and for anticipating further change [3], [4]. Analyzing food web dynamics is complicated in part because the communities we observe are likely not in “equilibrium” as we might have once expected [5]. There is increasing evidence that the structure of communities and the nature of species' responses to each other and to their environments are not static, but rather shift over time. In particular, anthropogenic pressures may be pushing communities further from equilibrium [6], with communities exhibiting a variety of non-equilibrium dynamics from smooth trends to abrupt step changes [7]. Changes in abiotic conditions of ecosystems can directly and indirectly affect food web structure [8]. Thus, food web models must account for diverse temporal changes in community dynamics. In some systems, while we may have a good understanding of average species interactions or effects of the environment on food web dynamics over key time periods, we may still lack important information about whether and how such dynamics changed over time in response to large shifts in the ecosystem.

Lake Washington, U.S.A., is an example of an aquatic ecosystem that experienced a series of well-described dramatic changes in its environmental conditions and plankton community in the mid-20^th^ Century. This time period included a regime shift from one of high nutrient loading from sewage inputs to one of increased water clarity, as well as temperature and species abundance changes [9]–[11]. The lake also experienced shifting regimes in terms of plankton community dominance. During the era of high sewage inputs, the lake experienced extensive nuisance algal blooms, especially of the cyanobacterium, *Oscillatoria rubescens*. Following sewage diversion, water clarity increased substantially [12]; subsequently, the influential grazer *Daphnia* established in the lake [11] and *Oscillatoria* effectively disappeared from the record. In more recent years, warming temperatures have caused phenological changes in phytoplankton and zooplankton [10], [13], [14]. What is unclear is how these changes in the plankton community and abiotic conditions affected interactions within the food web concomitant with the changing environment. Such shifts in plankton community interactions – such as weakening of grazer effects on phytoplankton, or increased competition among grazing zooplankton guilds – would have consequences for higher trophic levels in lakes, as plankton provides an important component of the energetic support for some lacustrine fish [15], including in Lake Washington [16]. Moreover, plankton community structure and indirect effects of herbivore-plant interactions can influence fundamental lake characteristics such as light, temperature and water clarity [17], [18]. In this paper, we introduce an extension of a well-used static food web model – a multivariate autoregressive (MAR) model [19]–[21] – to study Lake Washington's dynamically changing food web and ecosystem.

Over the last several decades, multivariate time-series methods have been used to estimate the strength and pattern of species interactions and the effect of abiotic drivers on communities [20], [22]. MAR models provide a locally linear approximation of non-linear stochastic multispecies processes. They have been particularly useful in aquatic ecosystems and for understanding plankton dynamics in part because of the tight coupling between plankton and their environment. MAR models have also become useful in broader aquatic food web analyses [23], [24], as they can incorporate multiple trophic levels and environmental drivers.

Prior implementations of MAR models have assumed that the interactions in the study system were unchanging over the time period encompassed by the data. This approach maximized the performance of parameter estimation given the properties of monitoring data, but only estimated the average interaction strengths over a time series. In contrast, if food web dynamics shift in response to changing drivers [25], then a better analytical approach would accommodate and capture this non-stationarity in modeling the food web. A suite of statistical methods can be applied to ecological time series to examine non-stationarity – such as shifts in abiotic conditions or periodicities – through time. Methods such as wavelets [26], [27], single-spectrum [28] and breakpoint analyses have been used in climatology and paleoclimatology, and have also recently been applied to ecological data [29], [30]. Such methods allow ecologists to see how abundances may be shifting [30] or how interactions among species may change over time in simple lab systems [29], but they do not provide a cohesive ecosystem approach to examining how integrated abiotic and biotic forces may change through time. In particular, food web responses to changes in the strength or nature of abiotic drivers would be predicted to cause cascading shifts in the interactions among many members of a food web, and may also feed back to how community members respond to other environmental drivers. Examining such a suite of interactions and drivers, however, would require a model that analyzes all the variables at once, and that allows estimation of such shifts through time.

A running or moving window approach is another tool that has long been used in other disciplines, such as finance, to examine non-stationarity in time series. In this approach, consecutive and overlapping subsets of time series – or windows – are analyzed individually to detect changes through time in a historical record [31], [32]. This approach has recently been used with univariate autoregressive models to develop leading indicators of regime change [33]–[35]. Here we offer an extension of the MAR model, which we term “moving-window MAR” (mwMAR), and we use it to examine a case of shifting species interactions and environmental effects on species through time. Our approach blends the community focus of the MAR model with the moving window approach of detecting historical changes in time-series data. We describe the mwMAR model and then apply the model to long-term monitoring data from Lake Washington, U.S.A., to show how interactions among dominant taxa of the plankton community shifted following sewage diversion. Because food webs show sensitivity to changes in their abiotic environment [6]–[8], we hypothesize that changes in the nutrient status, clarity, and dominant plankton taxa of the lake would cascade throughout the plankton food web, resulting in shifts in the direction and strength of community interactions, which would in turn affect community stability.

## Materials and Methods

### Model configuration

We estimated interaction strengths among phytoplankton and zooplankton guilds, environmental effects on phytoplankton and zooplankton abundance, plankton intrinsic growth rates, and plankton community stability in Lake Washington from 1962–1994 using multivariate autoregressive (MAR) models. MAR models are stochastic models describing changes in species abundance through time as a function of species interactions and environmental influences, while accounting for temporal autocorrelation in species abundances [20], [36], [37]. MAR models can also be used to estimate various metrics of community stability, such as return time to a stationary state following an ecosystem perturbation, or the distance away from a stationary state that an ecosystem can be pushed by a perturbation. Previous work has used MAR models to describe environmental effects on, and interactions among, lake phytoplankton and zooplankton [20], [22], [38], [39], effects of climate regime shifts on interactions among marine plankton [40], causes of estuarine fish declines [24], and effects of fishing on marine food webs [23]. Extended descriptions of MAR approaches to time-series data have been given previously [19], [20], [37], so we provide only a brief review of the model structure here.

MAR models are written in matrix form as:
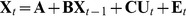
(Eq. 1)where, for *p* interacting species and *q* environmental covariates, **X** *_t_* is a *p*×1 vector of species abundances (here, natural log-transformed) at time *t*; **A** is a *p*×1 vector of constants, representing intrinsic per-capita growth rates; **B** is a *p*×*p* species interaction matrix, with off-diagonal elements describing inter-specific interactions, and diagonal elements describing intra-specific interactions (i.e., density-dependence); **C** is a *p*×*q* matrix with elements describing environmental effects on species abundance; **U** *_t_* is a *q*×1 vector of environmental covariates at time *t*; and **E** *_t_* is a *p*×1 vector of process errors at time *t*, representing environmental variation not otherwise accounted for in the model. **E** *_t_* is distributed as a multivariate Normal with mean **0** and a diagonal variance matrix ∑. Elements of **B** and **C** typically range from -1 to 1, with distance from 0 representing increasing negative or positive interaction strength. The diagonal elements of **B** typically range from 0 to 1, with values closer to 0 representing higher density dependence.

We also used MAR models to estimate community stability. Specifically, we estimated the rate at which the system returns to its stationary distribution following a disturbance by the maximum eigenvalue of the **B** matrix (that maximum eigenvalue is henceforth referred to as lambda, λ). Systems with values of λ closer to 0 are considered to be more stable because they tend to return to equilibrium conditions faster than systems with values of λ farther from 0 [21].

MAR models estimate mean intrinsic growth rates (captured by the **A** vector), community interactions (captured by the **B** matrix), environmental effects (captured by the **C** matrix), and community stability (captured by λ) across a given time series [20]. Here we use MAR models to quantify changes in interactions through time, by modeling community interactions for overlapping subsets of a time series, or moving “windows” of time, thereby estimating trends in MAR parameters. For a *p*×*n* matrix **X** of time series observations consisting of successive *p*×1 vectors **X**_1_, **X**_2_,…, **X** *_n_*, and a moving window of size *W*<*n*, we estimated MAR parameters within *n*-*W*-1 successive windows. These windows contained data from **X**_2_:**X** *_W_*_+1_, **X**_3_:**X** *_W_*_+2_,…, **X** *_n_*_-*W*+1_:X*_n_*. Note that the time series starts at *t* = 2 to allow for the lag-1 effect in Eq. 1. The output of the mwMAR analysis is a new time series of estimated MAR parameters.

### Lake Washington data and analysis

To investigate changes in interactions among zooplankton and phytoplankton guilds and the effects of environmental covariates in Lake Washington through time, we implemented the mwMAR approach using monthly plankton and environmental data from Lake Washington (Washington, U.S.A.) spanning 1962 to 1994 (396 timesteps; see Figure S1 for plankton time series). Our 33-year time series begins in the year of maximum sewage input (1962) when the lake experienced extensive nuisance algal blooms, especially of the cyanobacterium, *Oscillatoria rubescens*. Sewage diversion began the following year (1963), and was completed in 1968. Water clarity increased substantially by 1971 [12] and continued to improve through 1976, when the influential grazer *Daphnia* established in the lake [11] and *Oscillatoria* abundance decreased dramatically. Despite low abundances at times, and periods when they were not observe in samples, neither *Daphnia* nor *Oscillatoria* ever technically went extinct in Lake Washington. Before they begun to be observed at high abundances in 1973, *Daphnia* were observed every year but one (1971). Likewise, after their period of dominance ended in 1980, *Oscillatoria* continued (and continue) to appear in plankton samples, appearing in all but 3 years between 1980–1994.

The lake has additionally undergone significant warming throughout the historical record [10], which has altered the timing of zooplankton abundance cycles [14], [41]. Recent work, however, suggests species and nutrient (phosphorus) shifts related to the sewage effluent have had a stronger influence on the lake than shifts associated with warming [42]. These well-documented shifts in environmental drivers and plankton dynamics make Lake Washington an ideal ecosystem for evaluating the mwMAR model's sensitivity to non-stationary process. Indeed, the dominant environmental drivers and species interactions in Lake Washington are well-studied via observational [9], [12], experimental [43], [44] and traditional MAR approaches [39], [45], offering the necessary background to build informed community and environmental interaction matrices (**B** and **C** matrices, respectively).

For our analyses we aggregated physical, chemical and plankton community data, which were collected at various intervals, into monthly means. Previous analyses of the Lake Washington plankton community interactions identified a simplified food web containing species that demonstrated strong roles in structuring the community [39], [45]. We targeted the most strongly-interacting taxa of this simplified food web with the present analysis, to determine how the dominant interactions changed through time. While weak species interactions can be important in structuring food webs, we chose to focus on the dominant taxa and interactions as a first test of this new method. These taxa were pooled into four taxonomic groups: diatoms and green algae – “DG,” both palatable food for grazing zooplankton; *Oscillatoria* – known to suppress *Daphnia* [44]; *Daphnia;* and non-daphnid and non-cladoceran crustaceans – “NDC,” comprised of non-daphnid cladocerans, *Cyclops*, and *Diaptomus*. Group abundance data were log-transformed to better capture non-linearities [20]. A more complete description of the data is available in Hampton et al. [39], and the raw data are available in Appendix S1.

We included as covariates in the mwMAR model surface temperature and total phosphorus, because they were previously identified as the strongest environmental drivers of plankton abundance in the lake [39], [45]. However, rather than simply use temperature as a covariate by itself, we instead used the data to estimate (1) a mean monthly signal indicative of long-term seasonal forcing, and (2) monthly deviations from the mean to capture short-term anomalies (e.g., a particularly warm July) or long-term trends (e.g., an overall increase). To ease comparison of effect sizes across all environmental covariates, we standardized all covariate data to a mean of 0 and a standard deviation of 1.

For our environmental covariate matrix (**C**) we included *a priori* only biologically meaningful interactions based on established environmental relationships: we assumed total phosphorus could not directly affect *Daphnia* or other zooplankton taxa. We expected shifts in mwMAR coefficients to lag behind known dates of change in the biotic community, sewage diversion and water clarity because our moving window size (7 years) is much larger than the timescale of most known changes. We graphically present all data at the end year of the moving window; thus, in our figures, results based on data from 1963–1970 would appear on the x-axis at year 1970.

### Sensitivity analysis

The accuracy and precision of parameter estimates by the mwMAR model, as with other statistical methods, are sensitive to and affected by multiple factors, including food web configuration (i.e., the number of interacting species and covariates), window size, the variance structure of the process errors, and outliers in the data (see Appendix S2 for discussion and additional model validation). We conducted several sensitivity analyses to ensure such factors were not influencing the mwMAR model estimates. For example, the Lake Washington dataset is of high quality, and our outlier inspection showed no influence of outliers on the final results. In addition, because there is a tradeoff between precision of parameter estimates and accuracy of those estimates that is defined by window size, we conducted tests using simulated time series based on the Lake Washington food web configuration, to determine the appropriate window size for analysis of the Lake Washington dataset. In those simulations, the parameter estimation accuracy decreased sharply at window sizes smaller than 75 time steps (see Figure S1), and therefore we use a window size of 84 (the next factor of 12 larger than 75, given the monthly time step in the Lake Washington data). We also conducted simulations to determine what bias, if any, exist in parameter estimates during periods when a system is undergoing transition between states, for example between a eutrophic and clear-water state as was the case with Lake Washington. Last, to ensure that the mwMAR model was capable of capturing shifts in species interactions and environmental conditions outside of the Lake Washington case study, we fit mwMAR models to simulated time series with known interactions (see Appendix S2).

### Statistical programming

MAR and mwMAR modeling was done in MATLAB (2007, The MathWorks), using the open-source program LAMBDA ([46]; freely available from http://conserver.iugo-cafe.org/user/e2holmes/LAMBDA) with additional programming by the authors. The coefficients of the **A**, **B** and **C** matrices were estimated using conditional least squares (CLS), and confidence intervals around each coefficient were established using 2,000 bootstrapped data sets. Each bootstrapped data set was generated by creating random **E** matrices and fitting the rest of the parameters using CLS (see [21] for details).

## Results

**Figure 1:**
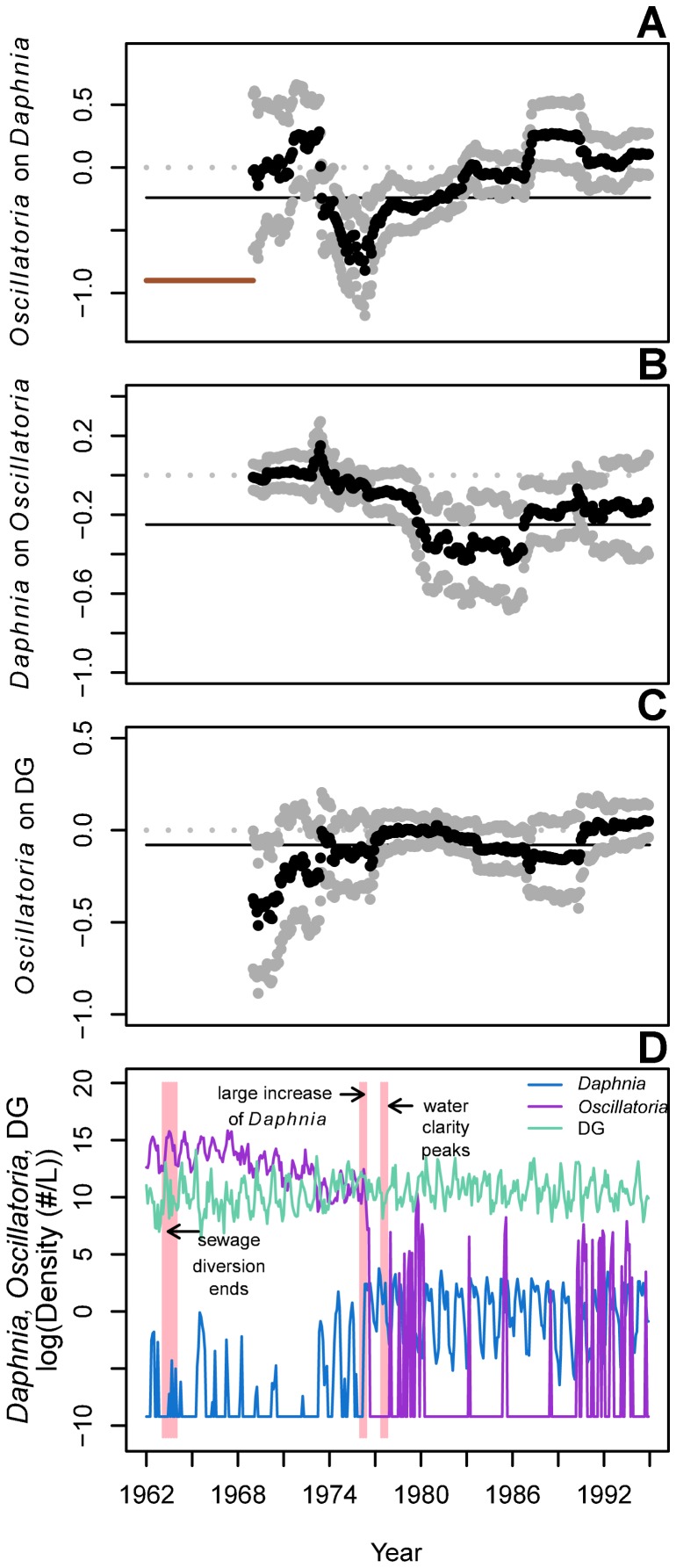
Shifting impacts of *Oscillatoria* on the Lake Washington plankton community. Effects of *Oscillatoria* on *Daphnia* (A); *Daphnia* on *Oscillatoria* (B); and *Oscillatoria* on diatoms/green algae, DG, (C) estimated by a mwMAR model using an 84-timestep window (indicated by solid red horizontal line shown in A). The mwMAR-estimated effect of *Oscillatoria* on NDC was non-significant. Estimates are shown with 95% upper and lower CIs. Grey dotted lines indicate a neutral interaction; solid black lines indicate the average interaction across the full time series, as estimated by a traditional MAR model. The raw time-series data are given in (D), with years of significant known changes shown in shaded vertical bars. All results are presented at the end year of the moving window.

**Figure 2:**
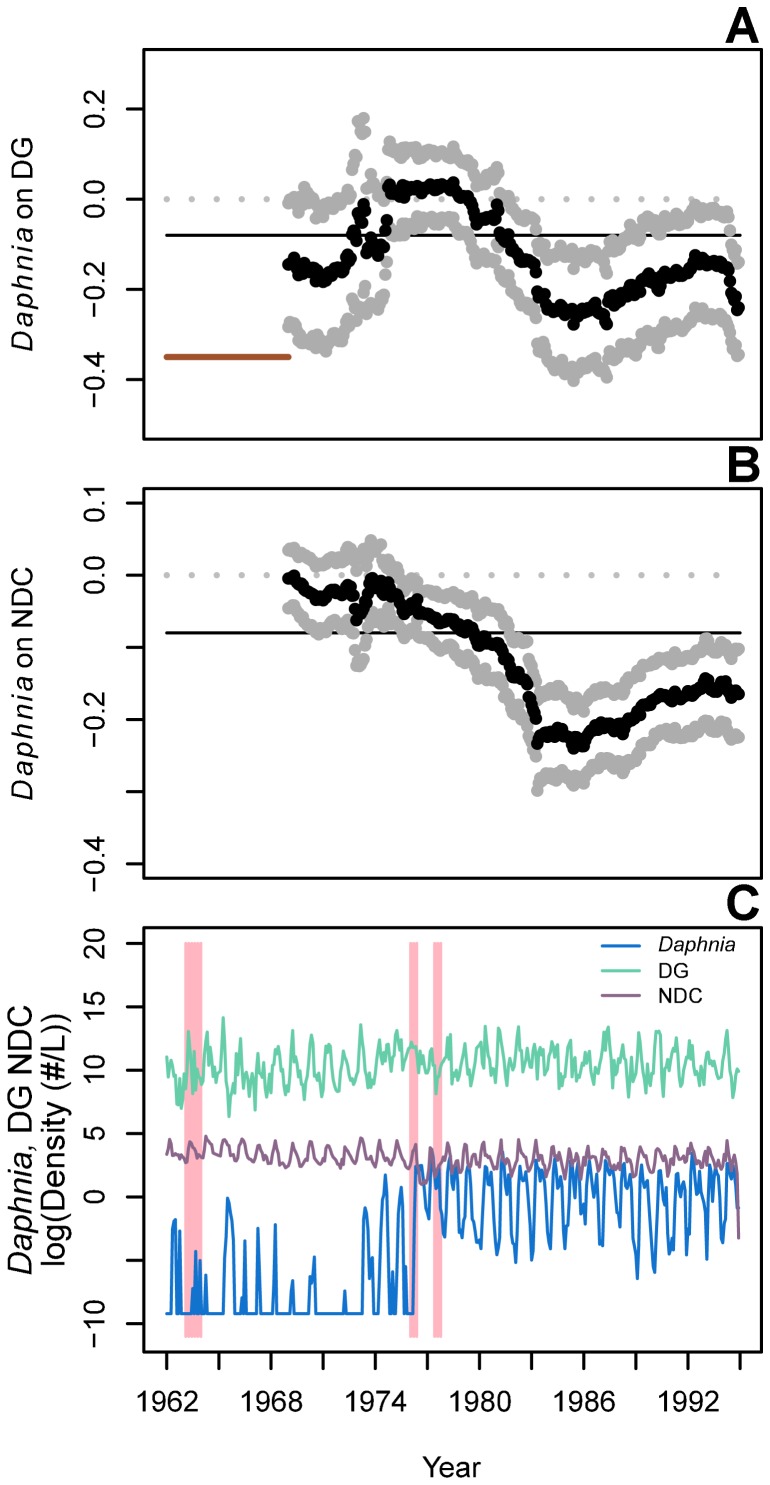
Shifting effects of *Daphnia* on the Lake Washington plankton community. Effects of *Daphnia* on diatoms and green algae, DG, (A) and non-daphnid cladocerans and non-cladoceran crustaceans, NDC, (B) through time as estimated by a mwMAR model with an 84-timestep window (indicated by solid red horizontal line in A). Estimates are shown with 95% upper and lower CIs. Grey dotted lines indicate coefficient values of 0; solid black lines indicate the average interaction across the full time series, as estimated by a traditional MAR model. The raw time-series data are given in (C), with years of influential known changes shown in shaded vertical bars. All results are presented at the end year of the moving window.

**Figure 3:**
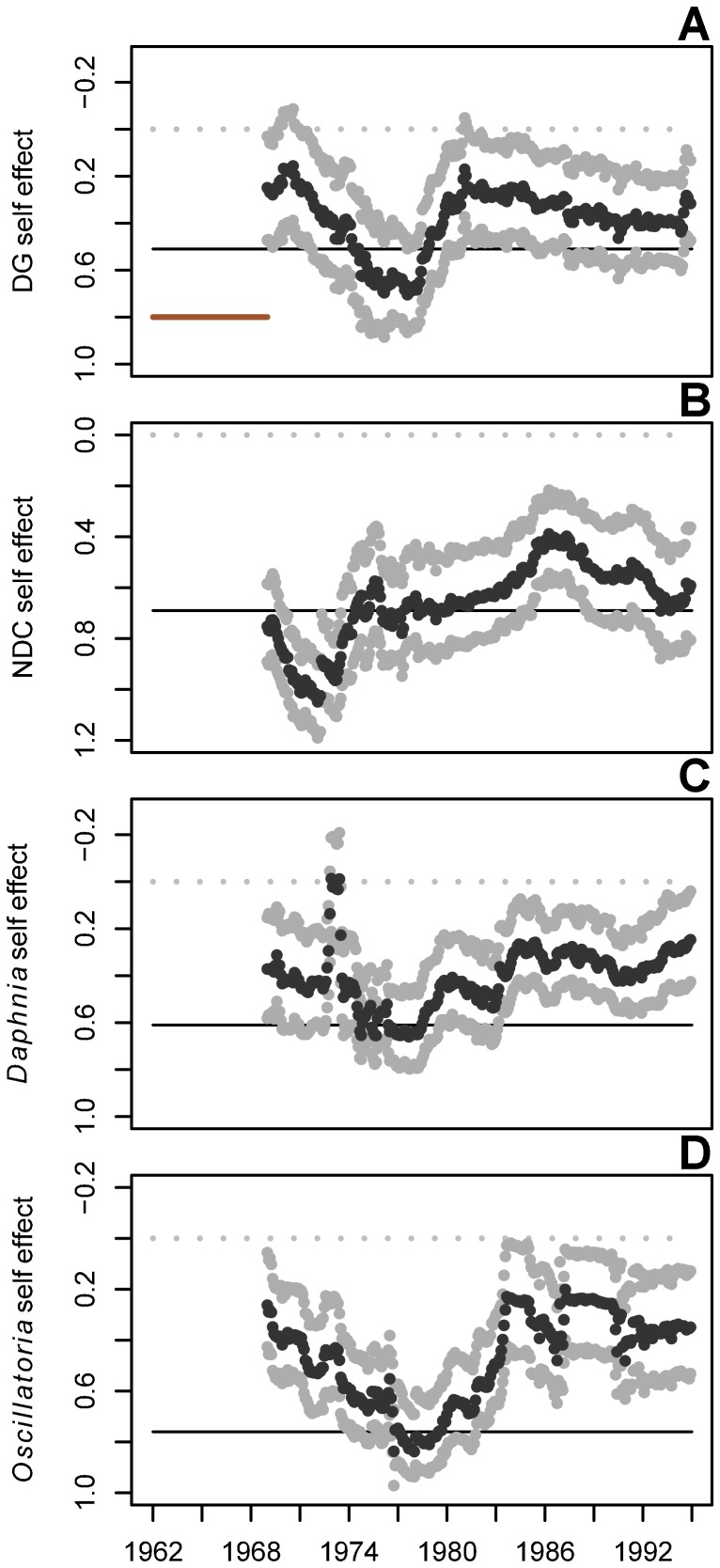
Shifting density-dependent effects of all plankton groups. Coefficients are estimated by a mwMAR model with an 84-timestep window (indicated by solid red horizontal line in A). Estimates are shown with 95% upper and lower CIs. DG  =  diatoms and green algae; NDC  =  non-daphnid cladocerans and non-cladoceran crustaceans. Grey dotted lines indicate coefficient values of 0; solid black lines indicate the average effect across the full time series, as estimated by a traditional MAR model. All results are presented at the end year of the moving window.

**Figure 4:**
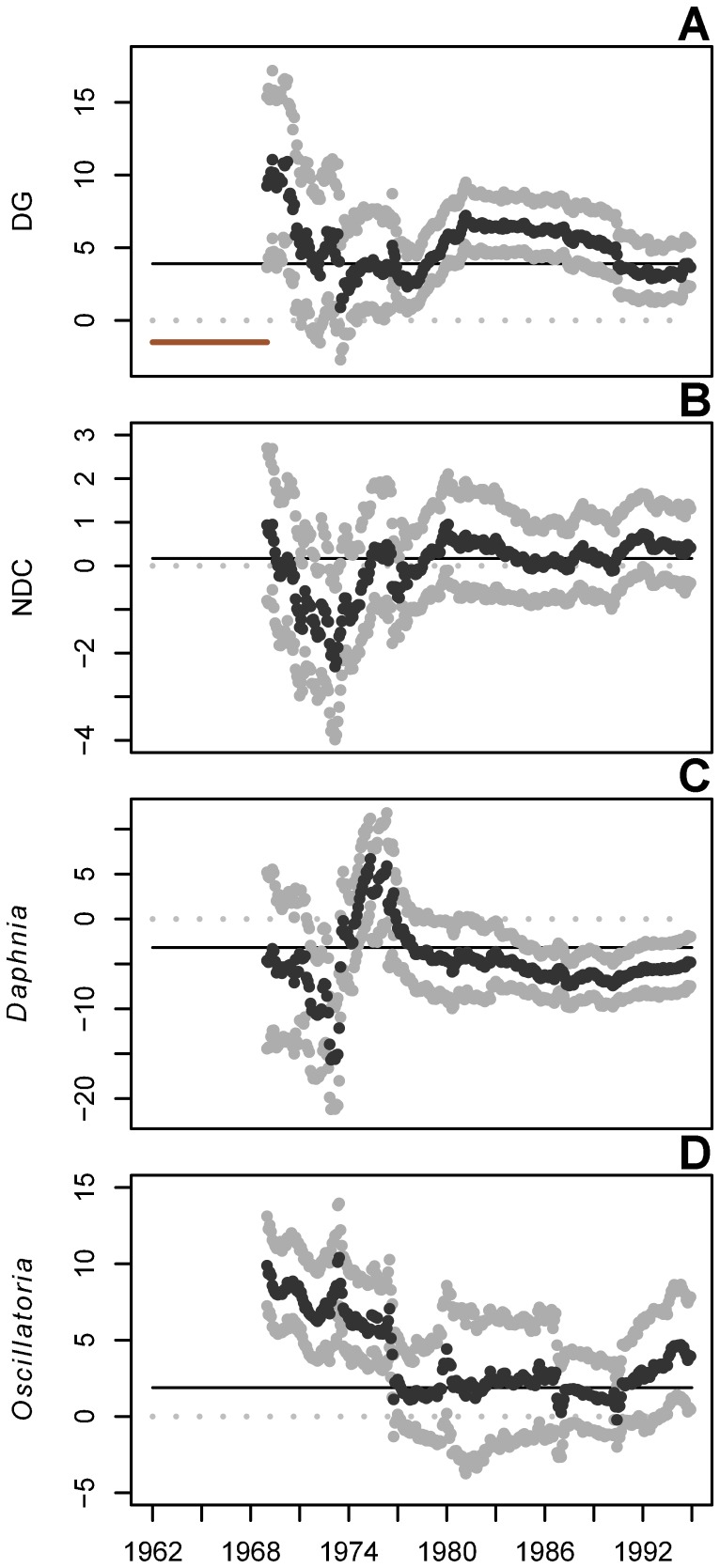
Intrinsic growth rates of Lake Washington plankton groups. Growth rates are estimated by a mwMAR model with an 84-timestep window (indicated by solid red horizontal line in A). Estimates are shown with 95% upper and lower CIs. Grey dotted lines indicate coefficient values of 0; solid black lines indicate the average rate across the full time series, as estimated by a traditional MAR model. DG  =  diatoms and green algae; NDC  =  non-daphnid cladocerans and non-cladoceran crustaceans. All results are presented at the end year of the moving window.

**Figure 5:**
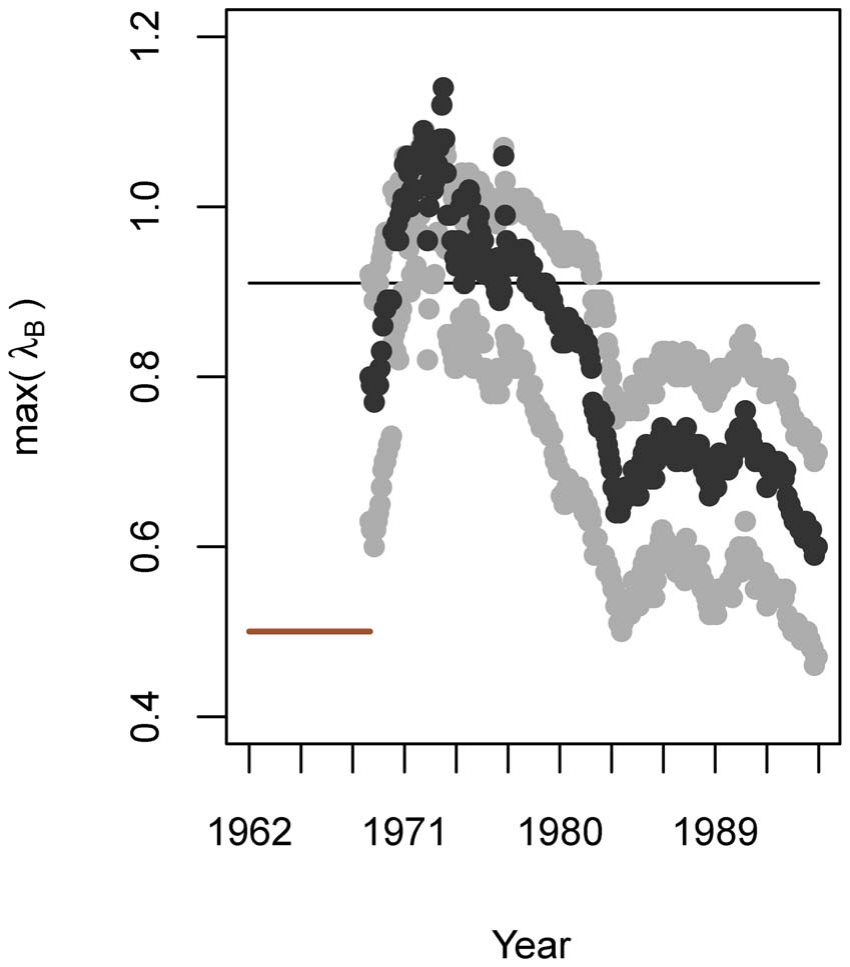
Shifting community stability. Stability is given by λ, the maximum eigenvalue of the community interaction matrix, as estimated by a mwMAR model using an 84-timestep window (indicated by solid red horizontal line). Estimates are shown with 95% upper and lower CIs. The grey dotted line indicates coefficient value of 0; the solid black line indicates the average stability across the full time series, as estimated by a traditional MAR model. Results are presented at the end year of the moving window.

The mwMAR approach revealed changes in interaction strengths in the Lake Washington plankton community between 1962 and 1994 (Figures 1–5; Figures S2–S3). For example, there were changes in the effects of *Oscillatoria* on *Daphnia* and diatoms and green algae (DG) coincident with the community composition shift during which *Oscillatoria* abundance decreased and *Daphnia* appeared (Figure 1, Table 1). In the period following the first appearance of *Daphnia* in Lake Washington, the effect of *Oscillatoria* on *Daphnia* became increasingly negative and was strongest in 1976 (Figure 1A). Following the decrease in *Oscillatoria* abundance, the negative effect of *Oscillatoria* on *Daphnia* weakened, and there was no significant effect of *Oscillatoria* on *Daphnia* from late in 1982 until the end of the time series. There was no effect of *Daphnia* on *Oscillatoria* (Figure 1B) until after the decline in *Oscillatoria* and increase in *Daphnia.* By 1980, the interaction coefficient became negative, weakened in the late 1980s, and returned to neutral after 1990. *Oscillatoria* also had a negative effect on DG in the beginning of the time series, and this effect disappeared by the mid-1970s (Figure 1C).

**Table 1:** Interaction ranges estimated by the mwMAR model for Lake Washington across all 84-timestep windows of data in the historical time series.

	Community Interactions				Covariate Effects		
	DG	NDC	*Daphnia*	*Oscillatoria*	Season	Temperature	Phosphorus
DG	0.15–0.70		−0.28–0.04	−0.52–0.05	−0.92–1.21	−1.25–0.10	−2.09–0.61
	(−0.09, 0.89)		(−0.40, 0.18)	(−0.89, 0.20)	(−2.75, 2.74)	(−2.33, 1.23)	(−3.99, 1.49)
NDC	0.00–0.18	0.39–1.05	−0.24–0.00	−0.08–0.09	−1.53–1.70	−0.59–0.73	*n/a*
	(−0.12, 0.28)	(0.22, 1.19)	(−0.30, 0.05)	(−0.17, 0.18)	(−0.77, 0.80)	(−1.23, 1.25)	
*Daphnia*	−0.32–0.46	0.08–1.46	−0.01–0.66	−0.82–0.29)	−1.38–2.72	−1.88–2.14	*n/a*
	(−0.67, 0.91)	(−0.65, 2.10)	(−0.21, 0.80)	(−1.18, 0.66)	(−4.40, 5.39)	(−3.61, 4.53)	
*Oscillatoria*	−0.44–0.21	−0.64–0.69	−0.43–0.15	0.20–0.84	−0.63–3.60	−2.02–1.45	−2.12–1.66
	(−0.91, 0.48)	(−1.16, 1.43)	(−0.68, 0.27)	(0.02, 0.97)	(−4.18, 6.74)	(−4.37, 4.22)	(−4.96, 3.96)

Empty cells reflect interactions not retained by the model. Parenthetical values represent ranges of 95% confidence intervals estimated for 2,000 bootstrapped data sets. Coefficients represent effects of variables in columns on variables in rows. DG: Diatoms/Green algae; NDC: Non-daphnid cladocerans and non-cladoceran crustaceans. *n/a* indicates a coefficient that was *a priori* excluded from the model.

The effects of *Daphnia* on other plankton groups in Lake Washington also varied through time (Figure 2). *Daphnia* had a negative effect on its main food source, DG, starting in the early 1980s, and the effect strengthened until the mid-1980s (Figure 2A). The effect remained negative, though slightly weaker, until the end of the time series. The effect of *Daphnia* on other zooplankton (NDC) also varied through time (Figure 2B). Similar to the *Daphnia*-DG interaction, after *Daphnia* established in Lake Washington, the effect of *Daphnia* on NDC became increasingly negative, reached its peak in the mid-1980s, then remained negative but weakened to the end of the time series.

Density dependence also varied through time for all plankton groups. Density dependence in DG decreased (i.e., the diagonal **B** matrix coefficient associated with DG increased) until after *Daphnia* established in the lake, after which density dependence increased (Figure 3A). Density dependence in the grazer group NDC increased steadily from the early 1970s until the late 1980s, after which it weakened until the end of the time series (Figure 3B). *Daphnia* density dependence increased from the time it established in Lake Washington until the end of the time series (Figure 3C). *Oscillatoria* density dependence weakened until its decline in abundance in the late 1970s, at which point it increased and held more or less steady from the early 1980s until the end of the time series (Figure 3D).

The MAR model also estimates density-independent intrinsic population growth (the **A** vector), and while many of the confidence intervals surrounding the **A** estimates overlapped zero for a portion of the time series, there were consistent trends in the estimates among different plankton groups (Figure 4). For all four plankton groups, there were three distinct periods of intrinsic growth rate estimates: (1) before regular appearances of *Daphnia* in Lake Washington (pre-summer 1973); (2) between when *Daphnia* began to make regular appearances, and when *Daphnia* established in the lake and *Oscillatoria* declined dramatically (summer 1973 – spring 1976); and (3) after the rise of *Daphnia* and decline of *Oscillatoria* (summer 1976 onward). During the first period, there was high variability and negative trends in all **A** estimates. During the second period, the DG growth rate was mostly constant (Figure 4A), both grazer groups growth rates increased (though some CIs overlapped 0; Figure 4B, C), and the *Oscillatoria* growth rate decreased (Figure 4D). During the final period, from the mid-1970s to the end of the time series, the growth rates of most groups were constant, except for an increase in the DG growth rate. Both *Oscillatoria* and NDC had growth rates equal to zero during this period.

Stability (λ) decreased sharply from the beginning of the time series, and the system was least stable (i.e., λ was at its maximum value) in the early 1970s (Figure 5). Following this nadir, community stability increased and reached maximum stability (i.e., the lowest λ value) at the end of the time series. Following bootstrapping, mean temperature had significant effects on all plankton groups. In contrast, very few effects of temperature anomalies or total phosphorus on plankton groups in Lake Washington were retained in the final mwMAR model (Table 1; Figure S3).

We assessed the fit of the best mwMAR model to the Lake Washington data, and found that fewer than 1% of correlations between model residuals and data were significant. We also tested the model assumption of normally-distributed errors by applying the Shapiro-Wilk test [47] to the residuals of the MAR fit to each data window (**E**, from Equation 1), with a Bonferroni-corrected alpha [48] to account for multiple null hypotheses. We rejected the null hypothesis of normally distributed errors in 65/312 windows for *Daphnia*, and in 217/312 windows for *Oscillatoria* (and in 0 windows for DG and NDC; Figure S4). These data windows for which the null hypothesis was rejected corresponded to periods in the time series when the abundance of each species was zero, i.e., the long one-sided tails in the data.

## Discussion

### Shifting plankton dynamics in Lake Washington

We hypothesized that the mwMAR would show shifts in the interactions among the major taxa corresponding roughly with known periods of change in Lake Washington (e.g., years of and around 1968–1971, and 1976). For example, it has long been hypothesized that the highly-abundant *Oscillatoria*, owing to its low palatability, inhibited *Daphnia* before *Daphnia*'s increase in Lake Washington in 1976 [11], during the period of time when the two species overlapped but *Oscillatoria* abundance was decreasing. These dynamics have been demonstrated experimentally [44], but our results are the first to corroborate this hypothesis using historical data. During the period of time between the peak in water quality (1971) and the dramatic increase in *Daphnia* abundance (1976) – the period of overlap between *Oscillatoria* and *Daphnia* and hypothesized inhibition of *Daphnia* by *Oscillatoria* – we found an increasingly negative effect of *Oscillatoria* on *Daphnia*. Once the mwMAR window included only dates following the large increase in *Daphnia* (i.e., 1976 and later), there was no detectable effect of *Oscillatoria* on *Daphnia*. The long, filamentous shape of *Oscillatoria* generally makes it inedible for *Daphnia*, which is one likely source of the negative per-capita effect estimated here during their period of overlap.

*Oscillatoria* also had a negative effect on diatoms and edible green algae, the main food source for *Daphnia* and other grazers in the lake. High intrinsic growth rates in edible phytoplankton estimated at the start of the time series decreased during the period when *Oscillatoria* was dominant. At the same time, density dependence in diatoms and green algae also decreased, suggesting inhibition in growth, possibly resulting from competition for limiting nutrients, or physical shading or toxic effects of excretions by *Oscillatoria*. Such inhibition of algae by *Oscillatoria* has also been demonstrated experimentally [44]. This apparent inhibition of phytoplankton by *Oscillatoria* rapidly decreased following an abrupt transition in the mid-1970s when the negative effect of *Oscillatoria* on DG decreased and disappeared (Figure 1). Coincident with these dynamics, the effect of *Oscillatoria* on *Daphnia* also weakened and the intrinsic growth rate of *Daphnia* increased from its minimum in 1972 to its peak in 1976 (Figure 4C). After 1976, *Daphnia*'s intrinsic growth rate decreased and density dependence increased (Figure 3C) as the *Daphnia* population increased in abundance. In addition, while the result was not significant (95% CIs overlapped zero), DG may have had a bottom-up positive effect on *Daphnia* after being freed from inhibition by *Oscillatoria*, in the latter half of the time series (Figure S2). Taken together, these results corroborate the hypothesis that the establishment of *Daphnia* following the improvement of water quality in Lake Washington was impeded directly and indirectly by the cyanobacterium *Oscillatoria*.

Grazers are known to inhibit cyanobacteria under some environmental conditions [49], and our analysis found a negative effect of *Daphnia* on *Oscillatoria* coincident with *Oscillatoria*'s decrease in abundance. In general, the frequency of cyanobacteria blooms is associated with the relationships between grazers and edible phytoplankton, such that when grazers and edible phytoplankton dynamics are stable (i.e., abundances do not undergo large, intrinsic oscillations), cyanobacteria are controlled by grazers [49]. These dynamics are often associated with phosphorus inputs to a lake. We observed a similar pattern in Lake Washington. As phosphorus inputs decreased, the grazing effect of *Daphnia* on edible phytoplankton increased concomitant with the inhibiting effect of *Daphnia* on *Oscillatoria* (Figures 1 and 2).

MAR coefficients have been shown previously to reflect changes in community dominance, when an increase in the abundance of one species or group coincides with a decrease in another [40], and therefore the negative effect of *Daphnia* on *Oscillatoria* may represent shifting dominance between the two taxa. The transition from *Oscillatoria* to *Daphnia* dominance was reflected in interactions among other plankton groups in the community. As the negative effect of *Oscillatoria* on *Daphnia* declined in the mid-1970s through to the early 1980s, and as *Daphnia* increased in abundance, *Daphnia* had stronger impacts on their main food source (DG) and competitors (NDC; Figure 2). At the same time, the strength of density-dependence (Figure 3) and density-independent growth rates increased for the grazing zooplankton groups (Figure 4), suggesting the release of the grazer community from inhibition by *Oscillatoria*. No previous work has shown an effect of *Oscillatoria* on other grazer groups beyond *Daphnia*, but the increase in population growth rates (**A** matrix elements) of the NDC group following *Oscillatoria*'s decline suggests a possible negative interaction.

The negative effects of *Daphnia* on their food and competitors weakened towards the end of the time series, apart from an intensified grazing effect of *Daphnia* on DG at the very end. One potential explanation for the weakened grazing effect at the end of the time series relates indirectly to the warming of the lake during this time. Between 1962 and 2002, the lake surface temperature increased by 1.4°C during the stratified months, and associated with this warming was an advance in the spring phytoplankton bloom by 19 days [10]. Most of the warming and spring bloom advance occurred in the period 1962-1994. The weakening of the effect of total *Daphnia* on the phytoplankton group during that period, in the present analysis, could be a reflection of shifts in species-specific phenology and grazing characteristics [14], [41].

The results presented here highlight opportunities to learn more from time series data about how species interactions shift with changes in the environment across ecosystem types, and how those changing food web dynamics are liable to affect community stability and resilience to further disturbance. Ecosystem-based approaches to management often include a focus on food web dynamics, but quantifying changes in species interactions, and how those changes map onto the environmental template, proves difficult. Linking shifts in species interactions to specific environmental drivers opens opportunities to focus efforts aimed at retaining resilience as ecosystems undergo rapid change.

### Community stability and environmental covariates

The Lake Washington system has undergone major shifts in chemistry and ecology that are reflected in community stability. The peak of instability occurred in April 1973, (a window that included data from May 1966 – April 1973; Figure 5). Values of λ greater than 1 indicate an unstable system [21], and here λ exceeded 1 for windows ending in November 1970 – November 1974, representing the period of time between December 1963 – November 1974, inclusive. This is the time period that included major ecosystem shifts: high nutrient levels, sewage diversion, and nutrient reduction; high *Oscillatoria* abundance followed by its decline; and the first rare appearances of *Daphnia*. By the time of *Oscillatoria*'s disappearance, maximum water clarity, and establishment of *Daphnia* in 1976, the community stability was increasing, and continued to increase until the end of the time series. Thus, the period of time the lake was undergoing the most substantial and dramatic shifts throughout the ecosystem, and before *Daphnia* gained a foothold, was the least stable period in the community as well.

We observed effects of monthly mean temperature on the abundance of all plankton guilds (Figure S3), which agrees with previous MAR analyses [39], [45], and with the MAR model estimated here from the whole Lake Washington time series (Table S1). Previous work has suggested that Lake Washington plankton phenology also responds to lake warming [10], [50], and that the relationships between temperature and plankton taxa are evidence of the potential influence of a warming lake on food web dynamics [39]. However, we found no significant effects of deviations from the long-term seasonal temperature patterns, suggesting that lake-warming effects are not detectable in the abundance of these plankton guilds.

### Caveats and considerations

Our results suggest moving-window MAR models may be useful in systems with sufficient time-series data for understanding shifting abiotic and biotic dynamics. As with all statistical methods, however, practitioners must consider possible caveats and issues in advance of and throughout analyses. The data and ecosystem considerations applicable to prior MAR model applications also extend to our moving-window approach. Users must have sufficient time-series data for valid parameter estimation, which varies depending on the time scale of interactions in the system and frequency of observations. The moving-window MAR model imposes the further consideration of having sufficient time-series data for multiple windows and surrounding the event(s) of interest. Importantly, bias in model estimates shrinks as the ratio between window size and system transition period increases, and users are cautioned to interpret model estimates during system transitions with consideration of such bias. However, the window could be configured for different purposes: made smaller to detect changes before they occur, or sized to optimize detection of a change in a particular state variable.

Applications of this method will benefit from *a priori* knowledge of ecological interactions and drivers in the modeled system to build a robust MAR model. In our analysis of the Lake Washington plankton community, we simplified the plankton community based on previous work that highlighted the strongest food web interactions and key environmental covariates [39]. However, Hampton et al. [39] also pointed out the importance of other plankton taxa in driving the dynamics of the dominant species in Lake Washington, such as *Cryptomonas*, picoplankton and non-colonial rotifers. Therefore, it is possible that additional food web dynamics contribute to the interaction coefficients observed here, which could be highlighted by future analyses. Furthermore, if the model failed to include an influential environmental driver of Lake Washington plankton dynamics, the model results might be erroneously interpreted: if one plankton guild responds negatively to an unmeasured environmental variable, and another guild responds positively, this might incorrectly be interpreted as a negative interaction between the two guilds. In the Lake Washington case, years of experimental work and field observations have identified environmental variables that are robust driving signals. In addition, preliminary, exploratory MAR model runs were performed to screen a broad suite of potential drivers on plankton time series data. The analyses here rely heavily on those two approaches to validation, and potential users are advised to similarly behave as ecological detectives.

Additionally, as with prior MAR approaches, users must invest time in simulation modeling that allows them to test how the approach is likely to work with data similar to theirs. Simulation of data from a model with similar parameters to the study ecosystem helps identify the appropriate moving window size and, thus, estimate the precision associated with future predictions of system change. Because much of the MAR approach is based on iterative fitting approaches, creating and testing simulation data sets from known parameter values with similar lengths, covariate and taxa numbers, and variance, is critical to interpreting knowledge gained from MAR models. For the moving-window approach, users should carefully examine the effect of window size on their simulation datasets (see Appendix S2 for an example analysis using simulated datasets). *A priori* knowledge or hypotheses related to the resolution of data and interactions as well as the strength and timing of the predicted shift should be considered during the process of simulation modeling. Comparison of the mwMAR output with whole time-series MAR estimates is useful in assessing when the broad confidence intervals estimated with the mwMAR model are potentially masking significant interactions.

## Conclusions

Ecologists have recently gained an appreciation for the need to develop methods based on the underlying hypothesis that many systems are rarely, if ever, stationary. Here we present a method that allows researchers and managers alike to examine long-term monitoring data and develop a dynamic record of shifting interactions and drivers. By calculating indirect and direct effects over time, and their changes, mwMAR allows researchers to understand how species invasions and extinctions, shifts in temperature and nutrient loadings, and other anthropogenic perturbations may cascade and feedback through food webs and ecosystems.

## Supporting Information

Figure S1 **Lake Washington plankton densities from 1962–1994.** Monthly means of densities for aggregated plankton groups used in mwMAR analyses. NDC  =  non-daphnid cladocerans; DG  =  diatoms and green algae.
(DOCX)

Figure S2 **Time series of all community interactions.** Interaction coefficients estimated for the Lake Washington time series with a mwMAR model, using an 84-month window. Figures show per-capita effects of plankton guilds in columns on plankton guilds in rows. Diagonal figures represent self-effects, or density-dependent effects on abundance.
(DOCX)

Figure S3 **Time series of environmental covariate effects.** Interaction coefficients estimated for the Lake Washington time series with a mwMAR model, using an 84-month window. Figures show the effects of covariates in columns on plankton guilds in rows.
(DOCX)

Figure S4 **Quantile-quantile plots of residuals for the** ***Daphnia*** **and** ***Oscillatoria*** **time series.** Shown are theoretical versus observed distributions of mwMAR model residuals for all windows where the Shapiro-Wilk test statistic was below the alpha value required to reject the null hypothesis of normally-distributed errors (61/1248 for *Daphnia*, 175/1248 for *Oscillatoria*, 0 for DG and 0 for NDC).
(DOCX)

Table S1 **Community and covariate matrix coefficients estimated by a MAR model for the full Lake Washington time series.**
(DOCX)

Appendix S1 **Lake Washington plankton and covariate data, 1962–1994.**
(CSV)

Appendix S2 **Moving-window MAR Model Testing.** Validation of the moving window MAR model approach, including accuracy of parameter estimation and estimation of bias during system transition.
(DOC)
