# Supplementary material for: Shifting Regimes and Changing Interactions in the Lake Washington, U.S.A., Plankton Community from 1962–1994
Source: PLoS One. 2014 Oct 22;9(10):e110363. doi: 10.1371/journal.pone.0110363 (PMC4206405; doi:10.1371/journal.pone.0110363)
Supplement: Figure S1 — Lake Washington plankton densities from 1962–1994. Monthly means of densities for aggregated plankton groups used in mwMAR analyses. NDC = non-daphnid cladocerans; DG = diatoms and green algae. (DOCX) [file pone.0110363.s001.docx]

**Figure S1. Lake Washington plankton densities from 1962-1994.**

Monthly means of densities for aggregated plankton groups used in mwMAR analyses. NDC = Non-Daphnid cladocerans; DG = diatoms and green algae.
