# Supplementary material for: Shifting Regimes and Changing Interactions in the Lake Washington, U.S.A., Plankton Community from 1962–1994
Source: PLoS One. 2014 Oct 22;9(10):e110363. doi: 10.1371/journal.pone.0110363 (PMC4206405; doi:10.1371/journal.pone.0110363)
Supplement: Figure S2 — Time series of all community interactions. Interaction coefficients estimated for the Lake Washington time series with a mwMAR model, using an 84-month window. Figures show per-capita effects of plankton guilds in columns on plankton guilds in rows. Diagonal figures represent self-effects, or density-dependent effects on abundance. (DOCX) [file pone.0110363.s002.docx]

**Figure S2. Time series of species interactions (B matrix).**

Interaction coefficients estimated for the Lake Washington time series with a mwMAR model, using an 84-month window. Figures show per-capita effects of plankton guilds in columns on plankton guilds in rows. Diagonal figures represent self-effects, or density-dependent effects on abundance.
