# Supplementary material for: Shifting Regimes and Changing Interactions in the Lake Washington, U.S.A., Plankton Community from 1962–1994
Source: PLoS One. 2014 Oct 22;9(10):e110363. doi: 10.1371/journal.pone.0110363 (PMC4206405; doi:10.1371/journal.pone.0110363)
Supplement: Figure S3 — Time series of environmental covariate effects. Interaction coefficients estimated for the Lake Washington time series with a mwMAR model, using an 84-month window. Figures show the effects of covariates in columns on plankton guilds in rows. (DOCX) [file pone.0110363.s003.docx]

**Figure S3. Time series of environmental covariate effects (C matrix).**

Interaction coefficients estimated for the Lake Washington time series with a mwMAR model, using an 84-month window. Figures show the effects of covariates in columns on plankton guilds in rows.
