# Supplementary material for: Shifting Regimes and Changing Interactions in the Lake Washington, U.S.A., Plankton Community from 1962–1994
Source: PLoS One. 2014 Oct 22;9(10):e110363. doi: 10.1371/journal.pone.0110363 (PMC4206405; doi:10.1371/journal.pone.0110363)
Supplement: Figure S4 — Quantile-quantile plots of residuals for the Daphnia and Oscillatoria time series. Shown are theoretical versus observed distributions of mwMAR model residuals for all windows where the Shapiro-Wilk test statistic was below the alpha value required to reject the null hypothesis of normally-distributed errors (61/1248 for Daphnia, 175/1248 for Oscillatoria, 0 for DG and 0 for NDC). (DOCX) [file pone.0110363.s004.docx]

**Figure S4. Quantile-quantile plots of residuals for the *Daphnia* and *Oscillatoria* time series.**

Shown are theoretical versus observed distributions of mwMAR model residuals for all windows where the Shapiro-Wilk test statistic was below the alpha value required to reject the null hypothesis of normally-distributed errors (61/1248 for *Daphnia*, 175/1248 for *Oscillatoria*, 0 for DG and 0 for NDC).
