# Supplementary material for: Shifting Regimes and Changing Interactions in the Lake Washington, U.S.A., Plankton Community from 1962–1994
Source: PLoS One. 2014 Oct 22;9(10):e110363. doi: 10.1371/journal.pone.0110363 (PMC4206405; doi:10.1371/journal.pone.0110363)
Supplement: Table S1 — Community and covariate matrix coefficients estimated by a MAR model for the full Lake Washington time series. (DOCX) [file pone.0110363.s005.docx]

**Table S1. Community and covariate matrix coefficients estimated by a MAR model for the full Lake Washington time series.**

|  | Community Interactions | | | | Covariate Effects | | |
| --- | --- | --- | --- | --- | --- | --- | --- |
|  | DG | NDC | *Daphnia* | *Oscillatoria* | Season | Temperature | Phosphorus |
| DG | 0.51 |  | -0.08 | -0.08 |  | -0.31 | -0.16 |
|  | (0.42, 0.59) |  | (-0.12, -0.04) | (-0.11, -0.04) |  | (-0.42, -0.2) | (-0.33, -0.02) |
| NDC | 0.07 | 0.69 | -0.08 | -0.03 |  |  | *n/a* |
|  | (0.03, 0.11) | (0.61, 0.76) | (-0.09, -0.06) | (-0.04, -0.01) |  |  |  |
| *Daphnia* | 0.16 |  | 0.61 | -0.24 |  | 0.44 | *n/a* |
|  | (0.01, 0.31) |  | (0.53, 0.68) | (-0.30, -0.18) |  | (0.26, 0.64) |  |
| *Oscillatoria* | -0.11 |  | -0.25 | 0.76 | 0.86 |  | 0.33 |
|  | (-0.22, -0.01) |  | (-0.31, -0.18) | (0.71, 0.81) | (0.63, 1.10) |  | (0.15, 0.57) |

Empty cells reflect interactions not retained in the final model. Coefficients represent effects of variables in columns on variables in rows. DG: Diatoms/Green algae; NDC: Non-daphnid cladocerans and non-cladoceran crustacean. *n/a* indicates a coefficient that was *a priori* excluded from the model.
