# Supplementary material for: Shifting Regimes and Changing Interactions in the Lake Washington, U.S.A., Plankton Community from 1962–1994
Source: PLoS One. 2014 Oct 22;9(10):e110363. doi: 10.1371/journal.pone.0110363 (PMC4206405; doi:10.1371/journal.pone.0110363)
Supplement: Appendix S2 — Moving-window MAR Model Testing. Validation of the moving window MAR model approach, including accuracy of parameter estimation and estimation of bias during system transition. (DOC) [file pone.0110363.s007.doc]

**Appendix S1. Model Testing.**

We conducted simulations to test the model performance specifically for the Lake Washington ecosystem. Users of mwMAR are advised to carefully test data and results, as with any method. In particular, we caution users to consider the influences of data outliers that may cause changes in coefficient estimates. As discussed in the main text, the Lake Washington dataset is of high quality, and our outlier inspection showed no influence of outliers on the final results. Additional model performance issues we address below are: sensitivity of coefficient estimates to window size; and accuracy of coefficient estimates relative to variance in the data. These same issues are concerns for users of other methods (for example, the influence of variance on ANOVA results), but owing to the complexity of mwMAR, these potential biases should be handled with special care. Simulations were conducted in R 2.13.1 . Basic MAR code is also available in the MAR1 package in R and could be modified for creating mwMAR.

Accuracy of parameter estimation by mwMAR model

To assess the influence of window size on precision and accuracy of parameter estimation by the mwMAR model and offer guidance about selection of window size, we estimated B and C coefficients by fitting the mwMAR model to simulated time-series data generated from known B and C elements, and 300 time steps in length. Importantly, we used interaction matrices that were different from the Lake Washington interaction matrices. We fit the mwMAR model to the same simulated time series using multiple window sizes, ranging from 25 to 200 time steps. We used root-mean-squared error (RMSE) of the estimated parameters as a measure of the model precision. Specifically, we calculated the RMSE based on the difference between the **B** matrix estimated by the mwMAR model (**B̂**) and the “true” **B** used in the simulation, such that

Equation 1.

where **E** = vec(**B̂** –**B**) and *k* is the total number of parameters in **B**. For each of the simulated food web configurations, we then calculated the mean RMSE across all windows of a given size to evaluate how accuracy improves with increasing window size (Figure A1). For the Lake Washington case, a sharp increase in inaccuracy occurred in windows smaller than 75 time steps, and therefore we used an 84-time step window (the next largest multiple of 12, as our data are in monthly increments) for our analyses.

Because we are using the mwMAR model to detect changes in the Lake Washington system and species interactions through time, we investigated how changes in species abundance were reflected in parameter estimates by the mwMAR model under different underlying causes. For example, could the mwMAR model discern between a change in species abundance owing to species interactions versus a change in abundance owing to changes in the environment?

We simulated abundance data for a 4-species food web based on Equation 1 (above), using 3 scenarios: a change in environmental conditions (i.e., the environmental input data included a positive trend in temperature; Figure A2); a change in species interactions; changes in both the environment and species interactions. All estimates were based on 50 random draws of the data. For all scenarios, the **U** vector (only one environmental covariate) remained constant, as

$\left[\begin{matrix} 0.8 \\ 0.001 \\ -0.03 \\ -0.8 \end{matrix}\right]$.

The **B** matrix for the scenario with changing environment was

$$\left[\begin{matrix} 0.7 & 0.00 & -0.2 & -0.1 \\ 0.0 & 0.65 & -0.1 & 0.0 \\ 0.2 & 0.1 & 0.6 & 0.0 \\ 0.2 & 0.1 & 0.0 & 0.5 \end{matrix}\right]$$

and in the scenario with changing species interactions, the **B** matrix changed at time step 150 from the above to

$\left[\begin{matrix} 0.6 & 0.00 & 0.1 & 0.2 \\ 0.0 & 0.7 & -0.1 & 0.0 \\ -0.1 & -0.1 & 0.3 & 0.0 \\ -0.3 & 0.01 & 0.0 & 0.5 \end{matrix}\right]$.

Under all changing conditions, the **B** parameters and stability (as maximum eigenvalue, or max(λ)) shifted as soon as the window entered the new phase of data (Figs. A2-A6). In the case of a change in species abundance owing to environmental change (Figure A3), after the window passed through the period of transition, during which it included both the pre-shift abundance data and the post-shift abundance data, the estimates (related to the affected species) returned to their original values. In the case of a true change in species interactions (Figure A4), the same transition period occurred, when the window included both pre- and post-shift data, but after the window passed through the transition period and included only data in the post-shift era, the estimated parameters reflected the new state. In both cases, stability showed a similar pattern to the **B** estimates. In the case where both environment and species interactions changed (Figure A5), the response of the **B** parameters resembled that of the scenario when just species interactions changed, but the stability pattern was more similar to the changing environment scenario. Thus, stability alone was not a good indicator of the underlying causes of changing state.

In all scenarios, the parameter estimates changed as soon as the window passed into a new phase, but the duration of the true transition to a new state was unknown. However, when we ran the mwMAR model backwards over the data from the changing environment/changing interactions scenario, we were able to better observe the point at which the transition occurs (Figure A6). In this case, the transition occurred over 1-2 data points, and therefore the shift in max(λ) is observable at the same point (time step 150) when analyzing the data forwards and backwards. If instead the transition to a new state occurred over a longer time period, there would likely be overlap between the two stability estimate lines. In addition, the period of transition between the two states is a function of the window size, such that smaller window sizes would result in shorter transition periods per length of time between states.

Estimate of bias during system transitions

We further conducted simulations to determine how much bias was introduced to the mwMAR model estimates by ecosystems undergoing state shifts, as in Lake Washington. We simulated time series data with a hypothetical community of 4 species and 1 covariate, 300 time steps in length, using the actual Lake WA variance/covariance matrix and known species interactions (B matrix) and covariate effects (C matrix). To assess the bias of the mwMAR model estimates of changing interaction strengths, we added to the simulated time series a transition in B at time step 150. We changed 2 interactions: B(3,2) from 0.3 -> -0.3; B(3,1) from 0.2 -> -0.2. To assess the bias of the mwMAR model estimates in a system undergoing abiotic regime change, we added a transition in the covariate between time steps 133-170 (i.e., over 36 time steps). The covariate underwent linear (plus random error) change from high to low value over 36 time steps. Bias in the B estimates was calculated as Bhat[i,j] – B[i,j], for window sizes 12, 24, 36, 48, 60, 72, and 84 time steps.

During the “transition period,” i.e., the time period when the moving window included dates during a state shift, the mean bias across all elements of B was not different from the mean bias during periods outside of the transition (Figure A7). When window length was equal to or greater than the transition period, mean bias inside the transition period was 0.12 (s.d. 0.15), and mean bias outside the transition was 0.12 (s.d. 0.18). The peak in bias occurred for most parameter estimates when the window was fully inside the transition, and began declining as soon as the window first left the transition, i.e., when estimates included time series data from the new state (Figures A8-A10). Likewise, stability decreased for the duration of the transition, and returned to a stable value after the window was fully in the new state (Figure A11).

**References**

Hochberg, Y. 1988. A sharper Bonferroni procedure for multiple tests of significance. Biometrika **75**(4): 800-802.

Ives, A., Dennis, B., Cottingham, K.L., and Carpenter, S.R. 2003. Estimating community stability and ecological interactions from time-series data. Ecol. Monogr. **73**(2): 301-330.

Scheef, L. 2013. MAR1: Multivariate autoregressive modeling for analysis of community time-series data. R package version 1.0.

Shapiro, S.S., and Wilk, M.B. 1965. An analysis of variance test for normality (complete samples). Biometrika **52**: 591-611.

Team, R.D.C. 2012. R: A language and environment for statistical computing. R Foundation for Statistical Computing, Vienna, Austria.

**Figures**

Figure A1. Root-mean-squared error (RMSE) as a function of window size used in mwMAR estimates of simulated time series based on 8 different food-web configurations. Symbols represent mean RMSE across all windows estimated in a 300-time step series. See text for simulation descriptions.


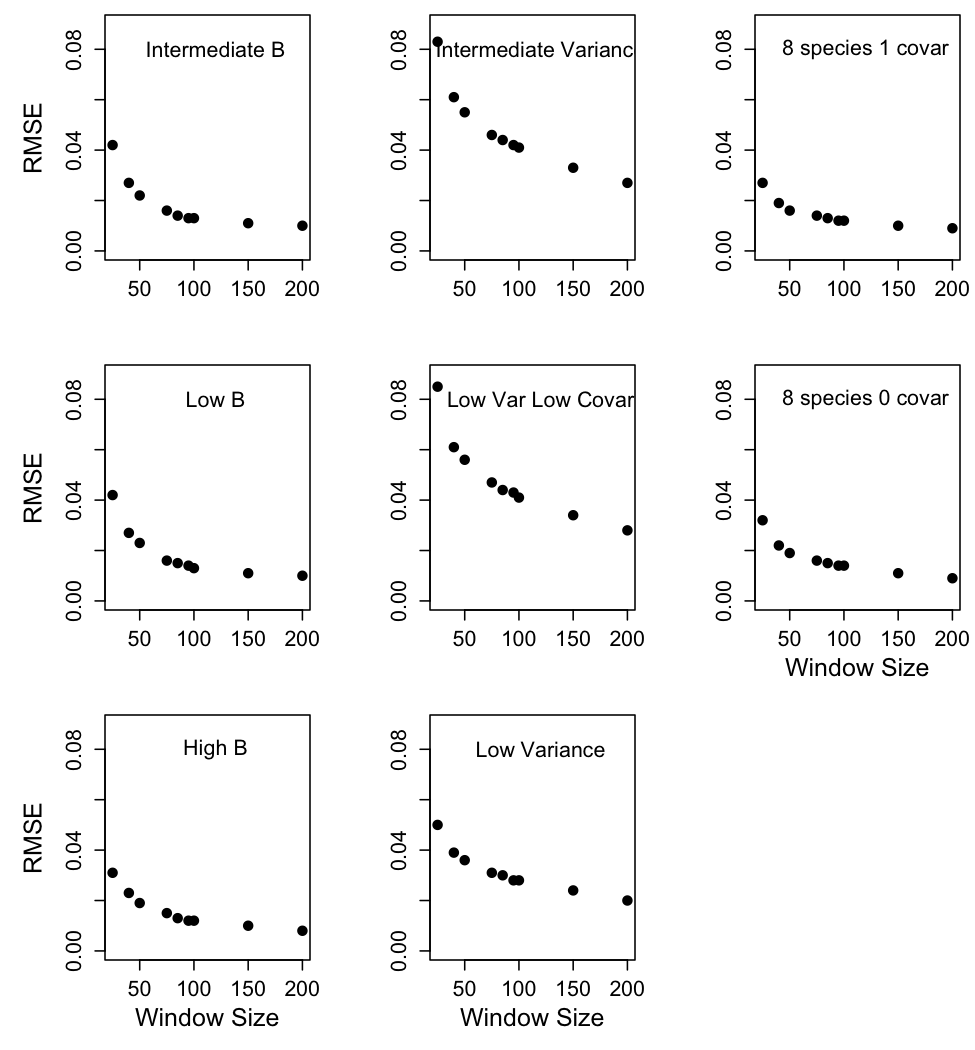


Figure A2. Environmental covariate data used in simulations to compare model response to changes in species abundance owing to changing environmental conditions versus changing species interactions. The red line shows the changing environmental conditions; the black line shows the constant environment scenario.


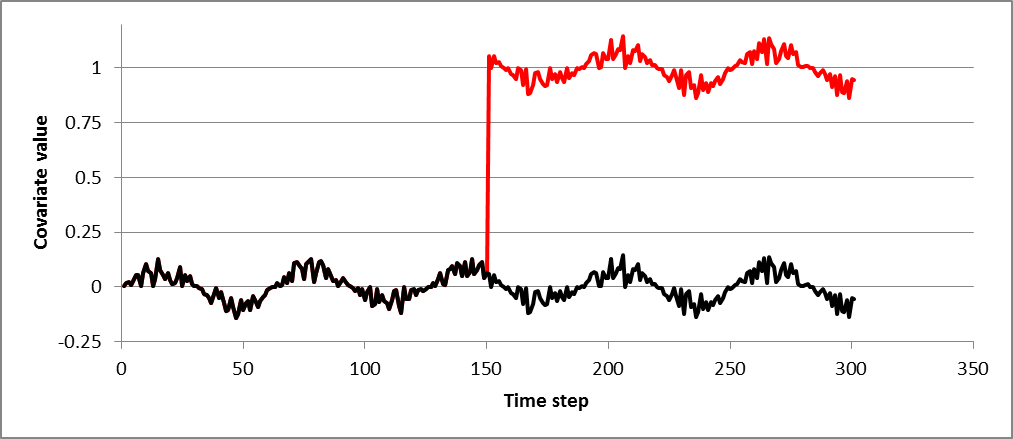


Figure A3. MwMAR parameter estimates using simulated data for a shift in environmental conditions (**U** matrix) at time step 150.


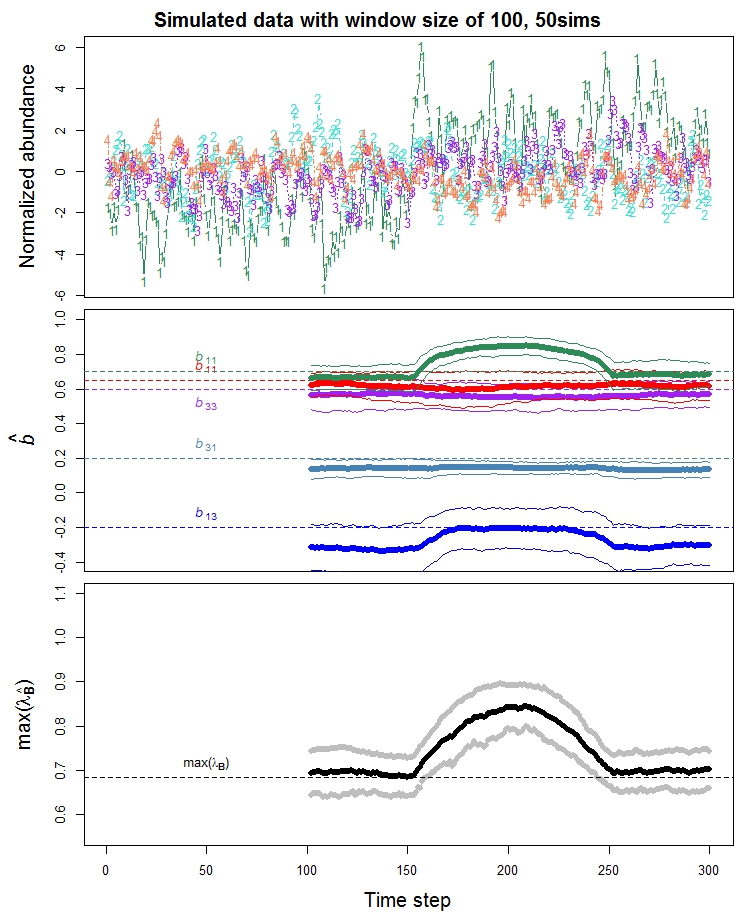


Figure A4. mwMAR parameter estimates using simulated data for a shift in community interactions (**B** matrix) at time step 150.


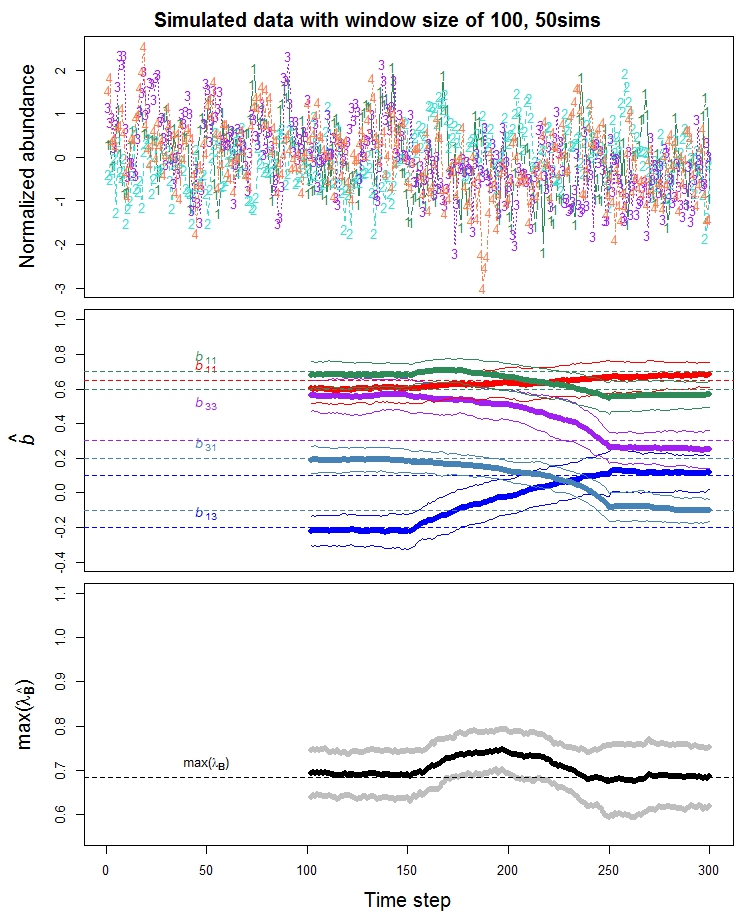


Figure A5. mwMAR parameter estimates using simulated data for a shift in environmental conditions and community interactions (**B** matrix) at time step 150.

.
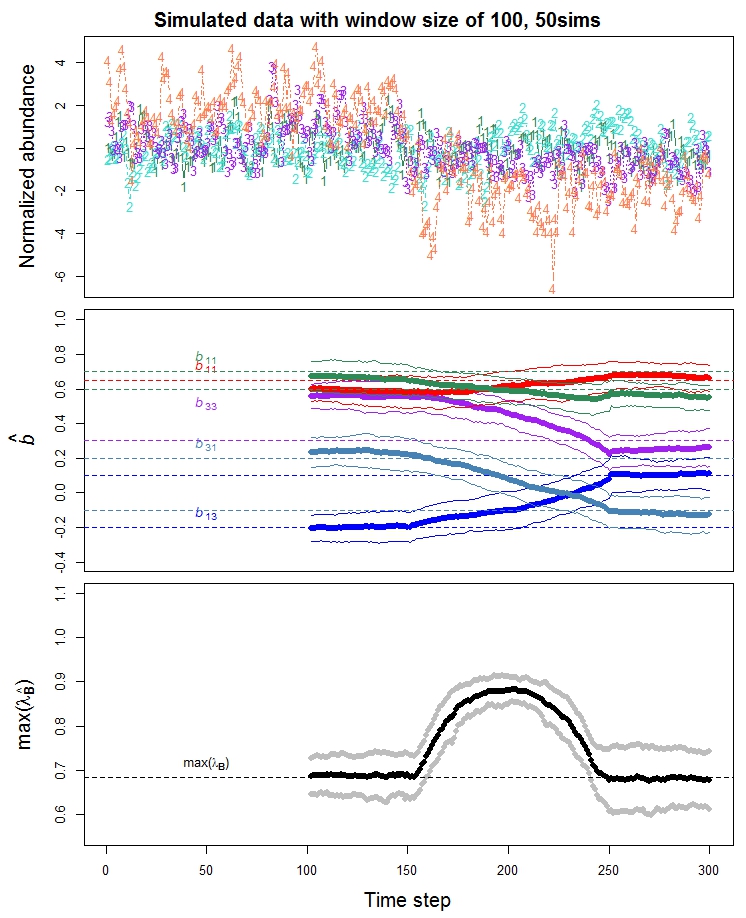


Figure A6. mwMAR parameter estimates using simulated data for a shift in environmental conditions (**U** matrix) and species interactions (**B** matrix) at time step 150, with the mwMAR model run forwards and backwards over the data.


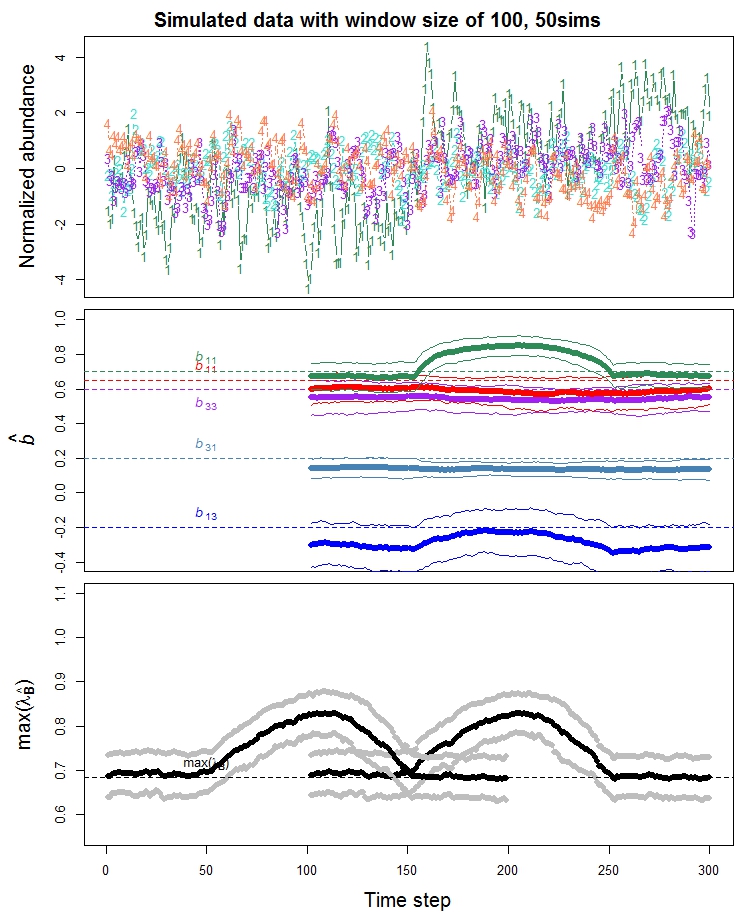


Figure A7.


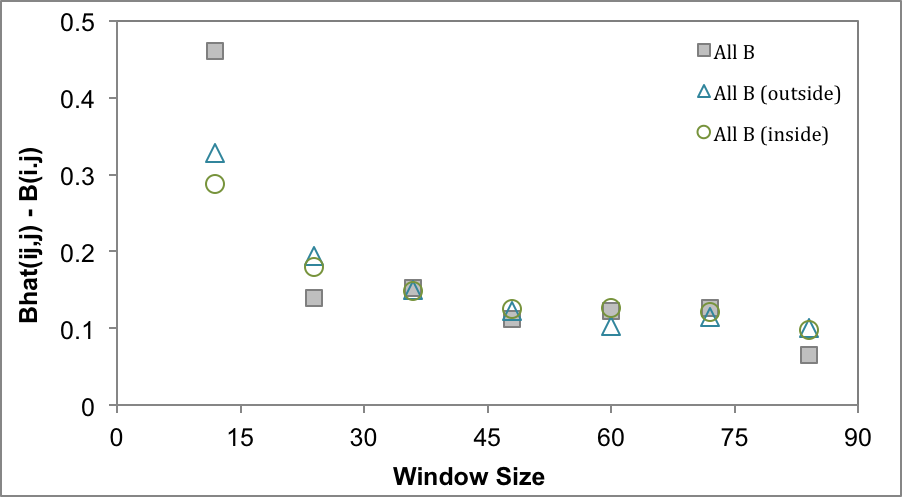


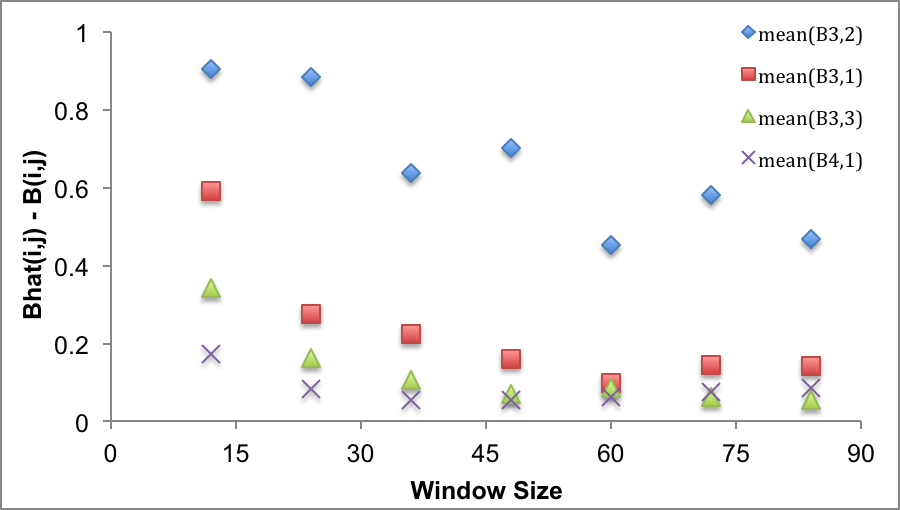


Figure A8. Time series of biases for a changing interaction (difference between estimated B3,2 and known B3,2) for different window sizes as the mwMAR model estimates interactions for a system undergoing a state transition (shown by dashed black lines). The full time series is 300 time steps; the transition is 36 time steps. The grey dashed lines represents the first estimate after the mwMAR window passes fully through the transition period. Window sizes are written at the far left of the time series.

Figure A9. Time series of biases for a changing interaction (difference between estimated B3,1 and known B3,1) for different window sizes as the mwMAR model estimates interactions for a system undergoing a state transition (shown by dashed black lines). The full time series is 300 time steps; the transition is 36 time steps. The grey dashed lines represents the first estimate after the mwMAR window passes fully through the transition period. Window sizes are written at the far left of the time series.

Figure A10. Time series of biases for a changing self effect (difference between estimated B3,3 and known B3,3) for different window sizes as the mwMAR model estimates interactions for a system undergoing a state transition (shown by dashed black lines). The full time series is 300 time steps; the transition is 36 time steps. The grey dashed lines represents the first estimate after the mwMAR window passes fully through the transition period. Window sizes are written at the far left of the time series.

Figure A11. Time series of stability for different window sizes as the mwMAR model estimates interactions for a system undergoing a state transition (shown by dashed black lines). The full time series is 300 time steps; the transition is 36 time steps. The grey dashed lines represent the first estimate after the mwMAR window passes fully through the transition period. Window sizes are written at the far left of the time series.
